# Hospital and Institutional News

**Published:** 1913-04-19

**Authors:** 


### HOSPITAL AND INSTITUTIONAL NEWS.

### THE YORK COUNTY HOSPITAL INQUIRY.

As we go to press we are informed that the promised public inquiry into the allegations made against the York County Hospital is being held at York on Friday, 18th instant, by Sir Cooper Perry. It will be remembered that the official announcement, which appeared in the York papers of March 19, stated that the members of the House Committee had "made application to the President of the Royal College of Surgeons of England to appoint someone who has knowledge of hospital management, and is at the same time free from all local influence," to "hold an impartial and independent inquiry." Our comment on the official announcement was that the above proposal could not be deemed satisfactory, if it meant a private investigation with closed doors. By the time that this issue is in the hands of our readers it will be evident exactly how the term "public inquiry" is being interpreted. It is absolutely essential that the inquiry should be public, in the full sense of the word, if the hospital is to maintain the respect of the public, who will not be satisfied unless every part of the case is discussed with the fullness and openness of a judicial tribunal.

### THE DUCHESS OF CONNAUGHT'S ILLNESS.

Of our princely houses there is none which stands higher in popularity and public esteem than that of T.R.H. the Duke and Duchess of Connaught. When, therefore, the British people heard of the successive attacks of illness which laid Her Royal Highness on a sick bed in Canada, genuine concern and sympathy were at once aroused. Recovering from the latest of these attacks, the Duchess returned to England, and the best advice was at once taken. The result was that operation was suggested, and Mr. Arbuthnot Lane was entrusted with its performance. In his hands the best possible result was a certainty, and the distinction which this selection confers upon the well-known Guy's surgeon is fully deserved. It is interesting, too, in view of the Duchess of Connaught's recent sojourn in Canada, to recall the fact that Mr. Lane's reputation stands higher across the Atlantic than that of perhaps any other British general surgeon now practising. At all events, the bulletins regularly issued have revealed nothing but the most satisfactory progress since the operation, which was evidently an extensive and severe one. Sir Bertrand Dawson, physician to the London Hospital and to the King, and Major Worthington, R.A.M.C., are in charge of the case with Mr. Lane. Her Royal Highness's progress has been so smooth that arrangements are already in contemplation for her removal to a warmer climate after the usual period of convalescence; and the Duke is shortly returning to his post in Canada with his recent anxieties, we trust, fully allayed.

### THE REMOVAL OF WESTMINSTER HOSPITAL.

The transference of Westminster Hospital from its old site opposite to the Houses of Parliament, so long discussed, has taken on a more practical phase by the grant of a three months' option on a provisional site in Clapham. It is understood that the site faces Clapham Common, is three acres in extent, and that its price is £9,000. Controversy as to its desirability in this case may be complicated by the fact that the erection of a hospital upon the site in question would mean the demolition of a group of fourteen old houses which are described as the work of Sir Christopher Wren. The "Victoria History of Surrey" is quoted as saying that the houses are "early eighteenth century, mainly three stories." "In two places," says the same authority, "the row is pierced by three-centred archways." The house built round the arch appears to have been a school at which Macaulay attended. We emphasise these points because a new site for a hospital presents sufficient difficulties of a technical kind, apart altogether from the business questions of cost and so forth, without being encumbered by possible opposition from interested minorities, as may be the case in this instance, on æsthetic and historical grounds. We presume, however, that Sir J. Wolfe-Barry, the chairman of the Westminster board of governors, has taken this factor of possible opposition into account before deciding to accept a grant for the option of the Clapham site.

### VERONAL AND THE POISONS SCHEDULE.

On Friday last week veronal and certain allied hypnotics contained in the Pharmaceutical Society's resolution became subject to the conditions imposed on the sale of substances governed by Part II. of the Poisons Schedule to the Poisons and Pharmacy Act, 1908. Nobody but a registered chemist and druggist may now sell it, and the word poison, together with the seller's name and address, must be placed on a label whenever the substances are sold without the written order of a medical practitioner.

### A HOSPITAL SECRETARY'S WARNING.

There is something more than the exposure of an impostor to be learnt from Mr. Arthur Watts' warning against "a man professing to be an ex-naval stoker, giving various names (Spencer, Durnsford, Dunscombe, Dunsford, Smurden) and addresses," who has been worrying subscribers to Queen Charlotte's Hospital for "a letter" for his wife. "He has obtained," says Mr. Watts, "several 'letters,' and it is known that some subscribers have, on his representing that he was out of work and in very reduced circumstances, given him money also. The 'letters' have not been presented at the hospital and the man is not known at any of the addresses he has given." This can almost be described as a new form of hospital abuse, which would not be possible were it not for the letter system. It can be said at once that this form of abuse is too infrequent to be used as an argument against a system which is always being criticised or defended. The present case, however, is only a flagrant instance of how lightly many hospital subscribers regard the responsibility which the possession of a letter confers. It is kinder, and more lazy, to give money or a letter than to inquire first as to the condition of the man who petitions for assistance. The letter system, though still regarded by many as a necessary evil, has involved the creation of inquiry officers, and too often has ended in giving away more money, judged by the cost per patient, than it brings in. The paradoxical justification of the system is seen in the commendable action of those subscribers who return their letters to the hospital for distribution.

### THE FEES FOR FREE CHOICE OF DOCTOR.

On the recommendation of the local medical committee for the county of Surrey, the following scale of fees in cases where insured persons have been allowed to make their own arrangements for medical treatment have been adopted: Attendance on the patient at the practitioner's residence, surgery, or dispensary, or visit to the patient's house, 2s. 6d.; special Sunday visit, 3s. 6d.; night visit (from 8 p.m. to 8 a.m.), 5s.; surgical operation requiring local or general anæsthetic, or case of abortion or miscarriage, 21s.; administration of general anæsthetics, 21s.; setting of fractures—(*a*) femur, 21s.; (*b*) others, 10s. 6d.; subsequent attendance at visit rates; reduction of dislocations—(*a*) hip, 21s.; (*b*) others, 10s. 6d.; subsequent attendance at visit rates. We may note also that the Surrey Insurance Committee has issued a new panel, from which ten doctors on the first panel have withdrawn, and to which six new names have been added.

### THE LONDON MENTAL HOSPITALS COMMITTEE.

The London Mental Hospitals Committee have elected Mr. E. G. Easton to be their chairman during the ensuing year. Mr. Easton, who is one of the Municipal Reform representatives of Fulham, is no stranger to mental hospital work. In the last Council he was a member of the committee, and showed his interest in its work by serving on eleven of its sub-committees. He was chairman of the Colney Hatch Sub-Committee, and was also a member of the sub-committees dealing with the Banstead, Bexley, Cane Hill, Hanwell, and Horton Hospitals. The vice-chairman of the committee will be Mr. H. V. Rowe, an Alderman, who was also a member of the committee in the last Council. Some difficulty is apparently being experienced in finding sufficient members to serve on the committee. Under standing orders the committee should consist of thirty to thirty-five members. Up to the present, however, only twenty-nine members have been appointed, and of these four have since resigned—Mr. H. E. S. Parsons, Mr. A. Cooper Rawson, Mr. R. L. Stuart, and Mr. T. W. Wickham.

### WARMTH AND LIGHT IN THE MORTUARY UNIT.

One important point about the mortuary unit is that the same care should be exercised over, and the same pains taken with, the ventilation of post-mortem rooms as in the case of the wards and operating theatres. To arrange for a system of ventilation independent of the heating is to court failure, although where open fires are provided the adjustment of a hot-water radiator may allow of an equal temperature without interfering unduly with the supply of fresh air and the exit of foul air. Artificial lighting of the post-mortem room and working rooms should always be electrical. In the post-mortem room, as well as in the working rooms, however, arrangements should be made for the supply of gas for heating purposes—such as Bunsen burners, gas ring for boiling solutions, etc. The post-mortem room is preferably lighted by roof lamps directly over the tables, side or wall bracket lights being avoided as far as possible.

### FOG AND LADIES' COMPLEXIONS.

A bold bid to gain the support of those ladies who are interested in securing the vote for their sex was made at a recent meeting of the Coal Smoke Abatement Society, when Dr. Arthur Latham remarked how much the appearance of women would be improved if they would only get politicians to take an active part in abating the smoke nuisance. Fair people who came to live in London, the speaker remarked, gradually became dark and the effect on their hair and complexions was very marked. We are afraid, however, that until the fog evil can be shown to produce a specific disease, for which tactically it would be advisable for a new name to be invented, people at large will never take seriously what they regard as the negative evils of absence of sunshine and impure atmosphere.

### THE HOSPITAL PATIENT AND THE ACT.

So very much in the great measure which will characterise historically the second decade of this century has, confessedly, been left to be moulded by experience, that it becomes most necessary to record, even piecemeal, just what that experience is turning out to be. As many instances as possible should be given, because the same results have not accrued everywhere, even in respects of primary importance. Thus the London Hospital apparently has found distinct reduction, even if not so large as was predicted, in the number of out-patients coming to the institution. But authorities elsewhere, as at large provincial infirmaries, have often another tale to tell: the congestion may not have visibly decreased at all, or even, as the result of that securely seated prestige which is rightly the pride of the modern hospital, have intensified at something more than the usual annual rate, and in the out-patient department it is noticeable that panel doctors are giving notes to patients to take up to hospital honoraries just about as often as they did before last July. This may be as much as anything else the pressure of lay opinion. Working-class men and women do not seem to mind how long they sit in the waiting-hall if at long last they get the workmanlike physical examination and the generally unhedged diagnosis. The *genius loci*, too, springing from the choice medicamentary array—the uniforms—the spaciousness—has them in spell. Why, if Mr. Lloyd George has put our affairs in order for us, has secured proper medical treatment for the proletariat in their own houses, do we hear such desperate and factious measures proposed as to annex half the existing medical beds in a large hospital for surgical cases? Nevertheless, that such a thing should be thought of is very significant in several respects. For the present purpose, enough to say that it may well be put on record that experience is all going to confirm the suspicion institutional authorities have had all along—namely, that, just as before, nearly all the difficult and dangerous work would come to them.

### LARGE BEQUEST TO WREXHAM INFIRMARY.

Mr. John Jones, of Grove Lodge, Wrexham, has bequeathed £50,000 to the Wrexham Infirmary, of which he was a vice-president. The institution, which is situated in Denbighshire, has about forty beds, and is probably one of the very few provincial hospitals of its size to receive so large a bequest. The provinces, happily for the voluntary system as a whole, have now created their own local reputation, so that the old anomaly of a wealthy country resident leaving his estate to institutions in London is much less frequent than it was. It will be interesting to learn from the Secretary, Mr. Frank Sisson, what his board propose to undertake with the large sum now placed at their disposal. Mining districts require specialised institutional accommodation, and the institutions which serve them are not always placed in so favourable a position as the Wrexham Infirmary has been by this bequest. A feature of the institution is its Samaritan Fund, which was started by the working-men to provide splints and other appliances for patients on leaving the hospital, which the institution itself could not afford to supply. The Samaritan Fund has also gone so far in the past as to present the infirmary with ward lockers and other theatre or ward furniture, which is a very extended interpretation of its objects, and this in addition to the assistance from it which patients have received.

### THE DIET OF THE SCHOOLBOY.

Not content with the amount of discussion created by their conference on diet in secondary schools last year, at which it will be remembered the schoolmasters, especially Mr. F. B. Malim, came out rather foolishly, the National Food Reform Association is organising another conference on the diet, cookery, and hygiene in elementary institutions. It is always rash to prophesy, but we should not be surprised if the elementary schools, in so far as they are boarding schools, proved equally unsound on the dieting of growing children. The institutional kitchen has become such an important feature in the hospital world, and has to cater for such numbers of patients, in addition to supplying many varied diets for special cases, that it seemed amazing to learn that the secondary schools were content with the abject confession that variety was impossible. Probably the reason why the school kitchen compares so badly as a rule with that of the institution for the sick is that in the latter, from the nature of the case, there can have been no temptation to economise, still less to make a profit out of the diet supplied. The school, on the other hand, has become very largely a commercial undertaking, and has even gloried in the tradition that the food should be meagre, or worse. What results, if any, the former conference achieved, except in showing how much the school might learn from the hospital kitchen, we do not know. But it is to be hoped that this second stirring up of interest in the subject will awaken the consciences of schoolmasters.

### NEW ASSISTANT SECRETARY AT CARDIFF.

The successor of Mr. F. Inch, who left the post of assistant secretary at King Edward VII.'s Hospital, Cardiff, to become secretary of the Walsall Hospital, is Mr. D. Rutter, of North Staffordshire Infirmary.

### A GREAT OPPORTUNITY AT CARDIFF.

Through the resignation of Dr. Herbert Vachell, the senior honorary physician to King Edward VII.'s Hospital, and through the death of Dr. Arthur Taylor, more than ordinary responsibility is thrown on those who have to fill the vacancies thus created. Now or never there is a great opportunity for the newly-appointed election committee to show that they grasp the principles that lie at the basis of a great and successful medical school. The men elected to fill the vacancies will be the men who may become teachers in the medical school, and possibly its future professors. It is, therefore, of the gravest importance that no other considerations whatever should be allowed to weigh against the highest qualifications obtainable in the men to be appointed to these posts. For the selected candidates will make or mar the new medical school, which, as it is the solitary medical school in Wales, will either attract to itself the highest prestige as a centre of medical education, or else may prove a failure in a double sense. No future appointments are, then, so important as these two, for once a standard has been set it is reasonably easy to maintain it, but the choice at the outset inevitably determines what the degree of the future standard shall be. In twenty years' time the reputation of the medical school will be what the election committee has been able and intelligent enough to make it to-day.

### AN AUSTRALIAN HOSPITAL SCANDAL.

The mismanagement of the "Queen's Memorial Infectious Diseases Hospital," otherwise known as the Fever Hospital, situated within the suburban area of Melbourne, which is controlled by the Metropolitan Municipal Councils, reached a climax last September, when, in consequence of certain statements made in the State Parliament, the

Government appointed Mr. V. Tanner, P.M., and a special board to inquire into the allegations against that institution. During the inquiry forty-four sittings were held and 156 witnesses were examined on oath. Mr. Tanner's voluminous report was published in the Melbourne papers on March 9. Amongst the charges which were held proved were the following: That the body of a patient was allowed to remain in the mortuary for ten days, and that no proper steps were taken to notify the relatives, and though the latter made daily inquiries by telephone, they invariably got the answer: "The patient is still improving." That the parents of another patient were informed by telephone that he was "just the same" some hours after he had died. That children sent to the hospital in a clean condition returned vermin infected, and also that juvenile patients were allowed to run about the hospital grounds imperfectly clad and barefooted, no action being taken by the medical superintendent when his attention was drawn to the practice. That dead bodies were carried on the shoulders of attendants past children playing in the grounds. That the secretary had not presented an annual report for some years. The probabilities are that effective steps will now be taken to prevent a repetition of such scandalous occurrences, which reflected discreditably on all concerned in the management of this latest addition to the list of Metropolitan hospitals in Victoria.

### NEW MEDICAL DIRECTOR-GENERAL OF THE NAVY.

Surgeon-General William May, C.B., has been appointed Medical Director-General of the Navy, by the First Lord of the Admiralty, in succession to Surgeon-General Sir James Porter, K.C.B. The new Medical Director-General was born in 1854, and entered the Navy as a surgeon in his twenty-fifth year. He studied at King's College Hospital and qualified M.R.C.S. in 1876 and L.R.C.P. a year later. He has seen active service in the Egyptian war of 1882, and two years later he took part in the expedition for the relief of General Gordon at Khartum. His attention to the wounded under fire was mentioned in despatches. He was made a C.B. in 1911, having gained promotion to his present rank in 1909, and during the present year he has been serving at the Royal Naval Hospital, Chatham. Sir James Porter, who is an honorary physician to the King, is an Aberdeen man, and was appointed to his present post in 1908.

### THE ANXIETY OF MILK PRODUCERS.

It will surprise nobody, with an ounce of experience, that great anxiety is stated to be agitating dairy farmers concerning the new Tuberculosis Order which comes into force on May 1. They are reported as saying that the production of milk will be so interfered with as to threaten the supply, and that inquisitorial inspections will drive dairymen to stock rearing in place of milk producing. Now we do not intend in a brief note to examine the value or otherwise of the new Order, but merely to remind institutional workers of two things. The first is that the argument of the dairy farmers is that which invariably comes from every industry and occupation to which inspection is extended, and is based simply on the dislike of human nature to any change of routine. The second is that institutions have a certain responsibility in this matter, for they have far too often been content with contracts containing "guarantees" of purity, without ever attempting to see that such clauses were enforced. For the action of the local authorities is sometimes sadly to seek in this matter.

### THE FATE OF THE DRAFT POOR-LAW ORDER.

In answer to several questions from Mr. Dawes on the subject of the draft Poor-Law Order Mr. Burns recently said: "The Departmental Committee appointed to consider the Poor-Law Orders with a view to their consolidation and amendment have not yet made their report to me, and consequently I am not in a position to say what their proposals are. I understand that some time ago the Committee invited the observation of the Poor-Law Unions Association and some other bodies upon certain of their proposals. As soon as I receive their report, which I understand will include a draft Order, I will give consideration to the various matters referred to by my hon. friend, including the suggestion that the draft Order shall be laid upon the table." This is studiously vague, but the lengthy criticisms which we have published, in which the draft Order is shown to be a timid compromise on matters of administration—on which its proposals, as far as they go, admit the principal of reform, while only half acting upon it—have evidently not been in vain.

### THE LATE SIR HENRY SWANZY.

As former president of the Royal College of Surgeons in Ireland, the late Sir Henry Swanzy, who died last week at the age of seventy, was one of the best known of Dublin surgeons. Educated at Dublin University, and oculist to the Lord Lieutenant from 1896 to 1898, in 1905 the Dublin University conferred on him the honorary degree of M.D. He was knighted in 1907, the year of his presidency of the Royal College of Surgeons, and was surgeon to the Royal Victoria Eye and Ear Hospital of the city. That his reputation extended beyond Ireland is evidenced by the fact that he was formerly president of the Ophthalmological Society of the United Kingdom. Among his many publications on ophthalmic subjects his "Handbook of Diseases of the Eye" has run into ten editions, and Dublin loses by his death not merely a distinguished surgeon but a personality and social figure.

### HERBALISTS AND PANEL SERVITUDE.

The rumour having gained currency that the scanty panels of the North were being recruited by unqualified medical practitioners, including herbalists "and other unauthorised persons," a question was asked in the House of Commons on Monday last. Mr. F. Hall, who asked whether arrangements could be made only with qualified practitioners, was assured by Mr. Robertson that this was so, and, further, that it was untrue that "in some instances Insurance Committees had accepted and placed upon their panels the names of herbalists." Ministerial denials are always useful as a matter of record, though the annals of the House at question time remain to prove that the art of evasion is one of the arts of speech not yet lost in a modern Parliament. The present reply, however, appears categorical and complete. We draw our readers attention to a letter in our correspondence columns from the secretary of the National Medical Guild in this connection.

### A LIVERPOOL HOSPITAL CHAIRMAN RETIRES.

We regret to announce that Colonel Robert Montgomery, acting on medical advice, is resigning the chairmanship of the Royal Liverpool Country Hospital for Children at Heswall, Cheshire. The resignation of few hospital chairmen can mean more than does Colonel Montgomery's to this institution. The peculiar individuality and scope of the Country Hospital has been alluded to more than once in our columns, but the resignation of its leading spirit may well be accompanied by a brief history of the growth of the idea for which it stands. In 1898 the possibility was discussed of founding a country hospital for children suffering from diseases, which, either because they were chronic cases or because the treatment was a very long one, the ordinary hospitals were unable to accept. In 1899 Colonel Montgomery accepted the chairmanship, formed a committee, and, thanks largely to his enthusiasm, a temporary hospital was opened with twenty beds. So successful did the experiment prove that in 1908 a new hospital was started at Heswall with eighty beds, to which two additional wards have now been added. Colonel Montgomery has been its keen champion from the first, and has had the gratification of seeing his institution's example followed in other places, and retires from the chairmanship at a time when the hospital has won an assured position, and a reputation that should make its financial future as free from anxiety as it is in the nature of the voluntary system to allow.

### THE DISMISSAL OF AN ATTENDANT.

Considerable feeling has been excited among mental hospital workers at Cardiff over the recent dismissal from Cardiff Mental Hospital of an attendant who also held the post of secretary of the local branch of the National Asylum Workers' Union. It appears that the attendant convened a meeting and sought signatures to certain petitions which alleged an offensive attitude in one of the deputy inspectors, and requested an allowance for rations to members of the nursing staff on their holidays. The question has been taken up as to whether the action of the ex-attendant amounted to a breach of the rules, which controversialists have since disputed, and whether the discipline of the establishment was likely to be interfered with. What the medical superintendent's view is may be judged from the action which was taken by the committee, though an unfortunate complexion has been given to the affair by such provocative phrases as "the victimisation of a trade-union official."

### THE EVOLUTION OF EPIDEMICS.

The Chadwick Public Lectures for 1913 are in course of delivery at the Royal Society of Medicine's House in Wimpole Street by Dr. J. T. C. Nash, medical officer of health and chief school medical officer for Norfolk. The first lecture dealt with the epidemics of the Middle Ages, and the second with those of the nineteenth century. The last lecture of the set will be delivered on Monday, April 21, at 8.15, and will embrace the epidemics of the present day. The Chairman of the Chadwick Trust, Sir William J. Collins, will preside; and admission is free. There will be epidiascope illustrations, and the Trustees are most desirous that Dr. Nash's lectures should be attended by members of the public as well as of the medical profession. Any further information will be willingly furnished by Mr. Aubrey Richardson, 8 Dartmouth Street, Westminster.

### THE GROVE HOUSE PROPERTY AT NORWICH.

It is interesting to learn that the proposals which were finally adopted in substitution of the suggested amalgamation scheme between the Norfolk and Norwich Hospital and the Eye Infirmary appear to be working well. The boards of the two institutions met and agreed to the purchase of a property known as Grove House, which is now being converted into an eye infirmary. This property, which was purchased by the Norfolk and Norwich institution, is let to the Eye Infirmary at a nominal rent, on condition that all the hospital's eye work be carried on there by the infirmary. The advantage of this co-operative scheme is that the annual expense of a separate block in the hospital grounds is saved, while the infirmary gains an extension at very small cost. The relation of special departments at general hospitals to special hospitals in their neighbourhood is not always so satisfactorily settled as this appears to have been, and both parties may be congratulated on it.

### ENCOURAGING CHILDREN TO SUPPORT THE HOSPITALS.

As an instance of what may be done by an enthusiastic head-master, we refer to the Broughton Astley Church of England School, the scholars of which have, through Mr. W. F. Dear, the head-master, sent a sum of £10 11s. 4d. to the Leicester Royal Infirmary from their ninth annual concert. Their effort discloses one of those latent sources of income which yet remain to be developed by the managers of up-to-date institutions.

### THE FUTURE OF MEDICAL EDUCATION.

The final report of the Royal Commission on University Education in London, which was appointed four years ago, has been issued as a Blue-book. The first unanimous point on which the Commission is agreed, as reformers will note with pleasure, is that the whole present organisation of the University is fundamentally defective, but how far reformers will agree with their proposals concerning the Faculty of Medicine remains to be seen. The Faculty is adopted by the Commissioners as the natural basis of University organisation, but they recommend that the preliminary sciences of physics, chemistry, and biology should no longer form part of the University curriculum in the Faculty of Medicine. Such studies, it is suggested, should be undertaken at secondary schools, and the passing of an examination in them alone would admit students to the Faculty of Medicine. As regards the intermediate sciences of anatomy and physiology and pharmacology the Commissioners recommend that these, together with pathology, should be provided for in as close proximity as possible to the hospitals where clinical teaching is given. As to the third part of the curriculum—medicine, surgery, and midwifery—they recommend that the teachers of subjects involving clinical instruction should be University professors, allowed only a limited amount of private practice, and not the practising physicians and surgeons who undertake the teaching to-day. Lastly, the Commissioners state that if all the University students, including those of Oxford and Cambridge now in London, were to be taught in University medical colleges, three colleges would be required, to which hospitals would be attached of sufficient size to provide for an annual entry of seventy-five students. The existing system of clinical teaching in London, by which each student holds clinical appointments and gains experience of the charge of patients, is warmly commended. With the wider questions of medical education we shall deal in a subsequent issue; this brief summary can merely conclude by saying that the estimated income required to organise a hospital medical college on University lines is £12,000 a year.

### THE LATEST CRITICISM OF "SECRET REMEDIES."

Before the Select Committee on the Sale and Advertisement of Patent and Proprietary Medicines, Mr. E. J. Parry, consulting and analytical chemist, took "Secret Remedies" to task on behalf of the Proprietary Medicines Section of the London Chamber of Commerce. He said that in the book entitled "Secret Remedies," published by the British Medical Association, there were such grave defects and inaccuracies that it fell short of its claim to disclose what such remedies contained and what they cost. He could give a list of about twenty drugs of the British Pharmacopœia, most of which were free from poisons, and all of which had started as proprietary and at one time secret medicines. When they had achieved success as such their value had been recognised by the medical profession, and they had been included in the British Pharmacopœia. The publication of formulæ would not diminish the sale of proprietary medicines, but would merely divert the sale from the original owner to imitators. As a result of the publication of "Secret Remedies" London street hawkers had sold the formulæ of remedies there printed for 1d. each. The speaker added that large numbers of preparations could not be correctly analysed chemically. Nor could, we fear, the claims made on their behalf: that is one reason why there should be no doubt as to the ingredients of patent medicines.

### THE LEGAL LIABILITY OF CONSUMPTIVES.

The legal liability for infection on the part of consumptive patients was the general problem raised by a recent suit in Ireland. A now deceased person and his friend called last year at the house of the plaintiff, a lodging-house keeper, and asked for lodgings. They stated in reply to her questions, suggested by the appearance of the former, that he was suffering not from consumption but from congestion of the lungs. The plaintiff therefore admitted them, and two days later a severe hæmorrhage occurred. The deceased was too ill to be moved for several weeks, and meantime three other lodgers in the house left. The plaintiff therefore sued the executor of the deceased, and the judge gave a decree for the full amount of the plaintiff's loss through the disinfection of the room, and a further sum for the loss of lettings.

### THIS WEEK'S DRUG MARKET.

Practically all the changes in the prices of drugs have been in a downward direction, which is quite unusual. The Drug market continues unusually quiet for the time of the year, which is rather difficult to explain in view of the undoubted increase in the consumption of drugs since medical benefit under the Insurance Act came into operation. As was anticipated, the price of cocaine hydrochloride has again been reduced; present quotations may be regarded as low, but there is no indication that buyers are in a hurry to make purchases, which may possibly be accounted for by the fact that there is an over-supply of raw material. Cod-liver oil still tends lower in price in the absence of demand; unless the fishing improves very considerably, however, a further substantial reduction can hardly be expected, as the results so far show that the yield of oil is less than half that at the same date last year. Cream of tartar is slightly cheaper. Calomel, corrosive sublimate, and other salts of mercury have been reduced in price by the makers. Carbolic acid tends lower in value.

### TO OUR READERS.

Contributions are specially invited from any of our readers to these columns. They should deal with topical subjects and news. They must be authenticated for the information of the Editor only. The minimum payment if published is 5s. There is no hard-and-fast-rule as to space, but notes of about twenty lines in length are preferred.

